# Whole exome sequencing identifies novel *USH2A* mutations and confirms Usher syndrome 2 diagnosis in Chinese retinitis pigmentosa patients

**DOI:** 10.1038/s41598-019-42105-0

**Published:** 2019-04-04

**Authors:** Tsz Kin Ng, Wenyu Tang, Yingjie Cao, Shaowan Chen, Yuqian Zheng, Xiaoqiang Xiao, Haoyu Chen

**Affiliations:** 1 Joint Shantou International Eye Center of Shantou University and The Chinese University of Hong Kong, Shantou, Guangdong, China; 2 Shantou University Medical College, Shantou, Guangdong, China; 3 Department of Ophthalmology and Visual Sciences, The Chinese University of Hong Kong, Kowloon, Hong Kong

## Abstract

Retinitis pigmentosa (RP) is a common phenotype in multiple inherited retinal dystrophies (IRD). Disease gene identification can assist the clinical diagnosis of IRD patients for better clinical management, treatment and counseling. In this study, we aimed to delineate and characterize the disease-causing mutations in Chinese familial and sporadic patients with initial diagnosis of RP. Four unrelated Chinese families and 118 sporadic RP patients were recruited for whole exome sequencing analysis. A total of 5 reported and 3 novel *USH2A* mutations were identified in four Chinese probands. The probands and their family members showed typical RP features and mild to severe hearing impairment, confirming the diagnosis of Usher syndrome 2 (USH). Moreover, 11 sporadic RP patients were identified to carry the compound heterozygous mutations in the *USH2A* gene, confirming the diagnosis of USH2. The patients carried the truncating mutations had a younger age of first visit than the patients carried only the missense mutations (*p* = 0.017). In summary, this study revealed 8 novel *USH2A* variants in Chinese familial and sporadic RP patients, assuring that whole exome sequencing analysis is an adequate strategy to facilitate the clinical diagnosis of USH from the sporadic RP patients.

## Introduction

Usher syndrome (USH) is an autosomal recessive disorder characterized by retinitis pigmentosa (RP) and bilateral sensorineural hearing loss, with vestibular dysfunction in certain patients^1^. USH is a leading cause of hereditary deaf-blindness with an estimated prevalence ranging from 3.2 to 6.2 in 100,000 people worldwide^2–4^. On the basis of the severity, the progression of hearing impairment and RP as well as the presence of vestibular dysfunction, USH can be divided into three clinical subtypes^5^. USH1, the most severe subtype of USH, is featured by the congenital hearing loss and vestibular dysfunction as well as the pubertal onset of progressive RP^6^. USH2, the most common subtype of USH accounting for more than 50% of all USH patients, is classified by the moderate to severe deafness and the pubertal onset of progressive RP, but with normal vestibular functions. USH3 patients exhibits progressive hearing loss, but has variable onset of RP and vestibular dysfunction^7^. Despite no cure for USH, cochlear implants can help the USH2 patients for better hearing function^8^. Yet, there is still no effective treatment to restore the retinal function in USH patients although gene therapy has been proposed^9^.

USH is clinically and genetically heterogeneous with 16 identified USH-associated genes^10^. Three genes are responsible for USH2: *USH2A* (usherin)^11^, *ADGRV1* (adhesion G protein-coupled receptor V1)^12^ and *WHRN* (whirlin)^13^. The *USH2A* gene is the major disease-causing gene for USH2; yet, it can also be relevant to non-syndromic RP or atypical USH^11,14^. The *USH2A* gene, located on chromosome 1q41, is coded for usherin, a transmembrane protein mainly produced in the photoreceptor layer of the retina, the hair cells in cochlea and the basement membranes of many tissues^15^. Different mutations in *USH2A* have been connected to non-syndromic autosomal recessive RP, apart from the typical USH2^16,17^. Precise diagnosis can be difficult, especially with atypical fundus appearance^18^. Disease gene identification can greatly assist the clinical diagnosis of individual RP patient for better clinical management, treatment and counseling.

In this study, we applied the whole exome sequencing (WES) analysis to hunt for the disease-causing mutations in four unrelated Chinese families and 118 sporadic patients with initial diagnosis of RP. In addition, the clinical correlation of the identified mutations was also analyzed.

## Results

### Whole exome sequencing analysis in familial RP patients

**Figure 1:**
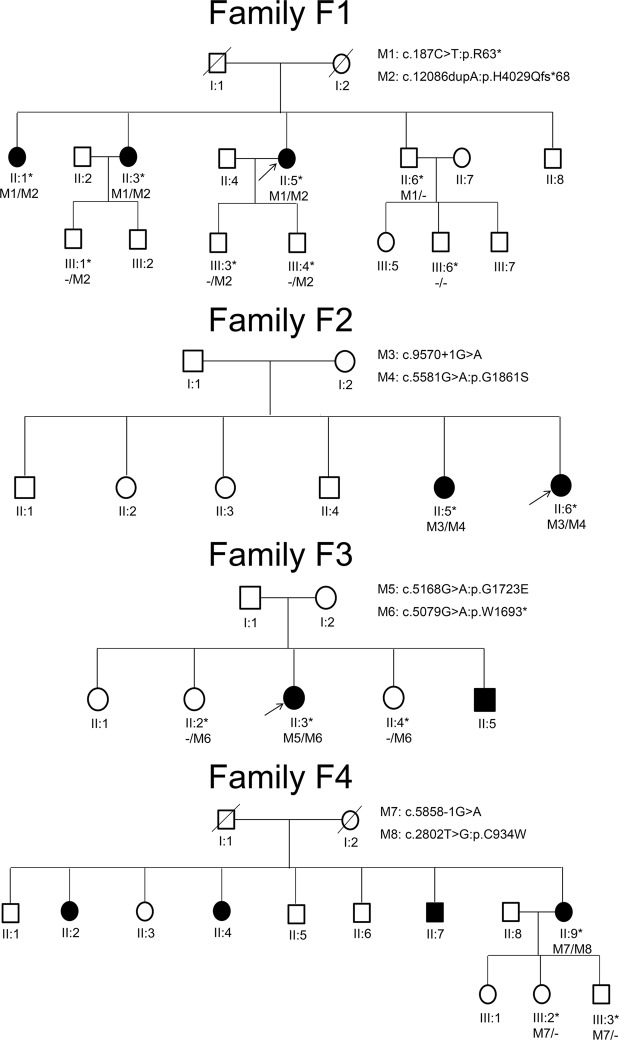
Pedigrees of the four recruited Chinese retinitis pigmentosa families. All the 4 recruited families initially diagnosed with RP followed the pattern of autosomal recessive inheritance. Squares and circles represent men and women respectively. The asterisk represents the participants’ blood were collected and the arrow indicates proband. The slash represents deceased person. Black: the affected members; White: the unaffected members.

Four unrelated Chinese families initially diagnosed with RP were recruited, following the recessive inheritance (Fig. 1). The proband of each recruited family was subjected to WES analysis. Each WES analysis resulted in a total of 70GB of sequence data, and 95.5% of sequence reads were originated from the exons, with a mean coverage of 100-fold. The total numbers of variants (SNPs and indels) of exons and splice sites identified were 23,774 for F1-II-6, 24,006 for F2-II-11, 23,499 for F3-II-6 and 23,911 for F4-II-15 (Supplementary Fig. 1). After filtering the synonymous, intergenic, intronic and common variants, the candidate variants of known RP genes were reduced to 2 for F1-II-6, 2 for F2-II-11, 5 for F3-II-6 and 3 for F4-II-15 (Table 1).

**Table 1:** Whole exome sequencing-identified variants in the probands of Usher syndrome-2 families.

Family	Gene	Mutation	Protein	Type	Inheritance mode	SIFT	Polyphen2 HDIV	Mutation Taster	CADD	MAF
F1-II-6	*USH2A*	c.187 C > T	p.R63*	Hetero	AR	T	N/A	D^3^	36	0
	*USH2A*	c.12086dupA	p.H4029Qfs*68	Hetero	AR	N/A	N/A	N/A	N/A	**Novel**
F2-II-11	*USH2A*	c.9570 + 1 G > A	Splice site	Hetero	AR	N/A	N/A	D^3^	15.17	5.44 × 10^−4^
	*USH2A*	c.5581 G > A	p.G1861S	Hetero	AR	D^1^	D^2^	D^3^	35	1.64 × 10^−4^
F3-II-6	*USH2A*	c.5168 G > A	p.G1723E	Hetero	AR	D^1^	D^2^	D^3^	24.9	**Novel**
	*USH2A*	c.5079 G > A	p.W1693*	Hetero	AR	T	N/A	D^3^	49	**Novel**
	*PRPH2*	c.367 C > T	p.R123W	Hetero	AD	D^1^	B	D^3^	14.31	1.50 × 10^−4^
	*EYS*	c.2980 C > G	p.P994A	Hetero	AR	T	P	D^3^	14.86	1.17 × 10^−3^
	*KIAA1549*	c.5653 C > T	p.R1885W	Hetero	AR	D^1^	D^2^	N	20.2	3.00 × 10^−4^
F4-II-15	*USH2A*	c.5858-1 G > A	Splice site	Hetero	AR	N/A	N/A	D^3^	19.71	5.45 × 10^−5^
	*USH2A*	c.2802 T > G	p.C934W	Hetero	AR	D^1^	D^2^	D^3^	16.44	2.51 × 10^−3^
	*CNGB1*	c.2681 G > A	p.R894H	Hetero	AR	D^1^	D^2^	D^3^	33	5.12 × 10^−5^

AD: autosomal dominant; AR: autosomal recessive; B: benign; D^1^: deleterious; D^2^: damaging; D^3^: disease-causing; Hetero: heterozygous; MAF: minor allele frequency in East Asian population; N: polymorphism; N/A: not available; P: possibly damaging; T: tolerated.

**Figure 2:**
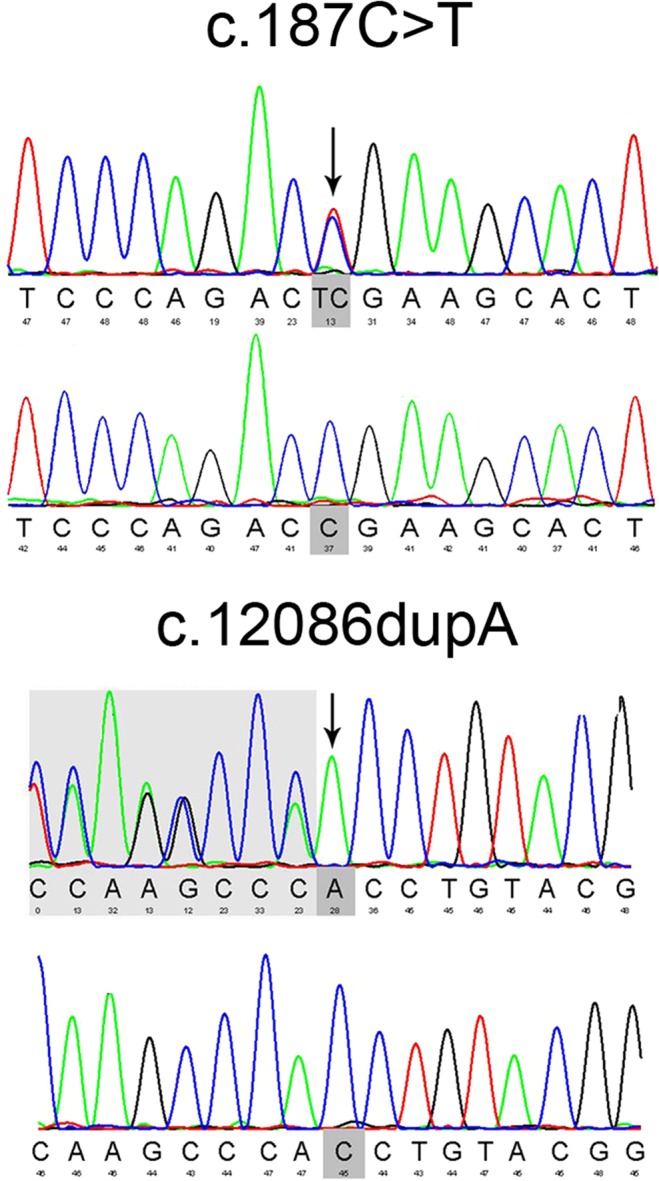
Sanger sequencing confirmation of the identified *USH2A* mutations from the whole exome sequencing analysis in the recruited Chinese retinitis pigmentosa families. Sanger sequencing validation of the *USH2A* mutations (c.187 C > T and c.12086dupA) identified by the WES analysis in USH family F1.

For the family F1, two heterozygous variants in *USH2A* gene were identified in all three patients (F1-II-2, F1-II-4 and the proband: F1-II-6): a nonsense variant c.187 C > T (p.R63*) in exon 2 and an insertion frameshift variant c.12086dupA (p.H4029Qfs*68) in exon 62. The identified variants has been confirmed by Sanger sequencing in these patients (Fig. 2). Their unaffected brother (F1-II-7) only carried the nonsense variants (c.187 C > T; p.R63*), whereas the three unaffected third-generation male subjects (F1-III-5, F1-III-8 and F1-III-9) only carried the insertion frameshift variant c.12086dupA (p.H4029Qfs*68), which was transmitted from the affected patients (Fig. 1 and Table 1). The c.187 C > T (p.R63*) variant has been previously reported^19,20^. The c.12086dupA (p.H4029Qfs*68) *USH2A* variant was neither found in 1000 genome, gnomAD and dbSNP nor previously reported, suggesting that it is a novel mutation. Furthermore, the c.187 C > T (p.R63*) and c.12086dupA (p.H4029Qfs*68) variants were also not found in 200 control subjects from our cohort. The c.187 C > T (p.R63*) and c.12086dupA (p.H4029Qfs*68) variants should be the causative mutations for the USH2 family F1.

For the family F2, two heterozygous variants in *USH2A* gene were identified in the two patients (F2-II-10 and the proband: F2-II-11), a non-synonymous variant c.5581 G > A (p.G1861S) in exon 28 and a splice site variant c.9570 + 1 G > A in intron 48 (Table 1). These two variants have been previously reported^20,21^. The c.5581 G > A (p.G1861S) and c.9570 + 1 G > A variants should be the causative mutations for the USH2 family F2.

For the family F3, two heterozygous variants in *USH2A* gene were identified in the proband (F3-II-6), a nonsense variant c.5079 G > A (p.W1693*) in exon 25 and a non-synonymous variant c.5168 G > A (p.G1723E) in exon 26 (Table 1), both of which are novel variants. The unaffected members (F3-II-4 and F3-II-8) only carried the nonsense variant c.5079 G > A (p.W1693*). In addition, non-synonymous variants in *PRPH2* (c.367 C > T; p.R123W), *EYS* (c.2980 C > G; p.P994A) and *KIAA1549* (c.5653 C > T; p.R1885W) genes were also found in the proband (F3-II-6); yet, these variants did not co-segregate with the phenotype in the pedigree. The c.5079 G > A (p.W1693*) and c.5168 G > A (p.G1723E) variants should be the causative mutations for the USH2 family F3.

For the family F4, two heterozygous variants in *USH2A* gene were identified in the proband (F4-II-15), a non-synonymous variant c.2802 T > G (p.C934W) in exon 13 and a splice site variant c.5858-1 G > A in intron 30 (Tables 1 and 2). Both variants have been previously reported^16,17^. The unaffected children (F4-III-34 and F4-III-35) only carried the splice site variant c.5858-1 G > A. Besides, non-synonymous variant in *CNGB1* gene (c.367 C > T; p.R123W) was also identified in the proband (F4-II-15) and his unaffected son (F4-III-35), but the *CNGB1* variant did not co-segregate with the phenotype in the pedigree. The c.2802 T > G (p.C934W) and c.5858-1 G > A variants should be the causative mutations for the USH2 family F4.

**Table 2:** Clinical manifestations of the family members in the Usher syndrome-2 family F1.

Members	II:2	II:4	II:6	II:7	III:5	III:8	III:9	III:11
Consult age (Y); gender	58; F	54; F	53; F	50; M	32; M	27; M	25; M	27; M
Onset age (Y); presenting symptom	12; nyctalopia	14; nyctalopia	10; nyctalopia	NA	NA	NA	NA	NA
Hearing loss (R/L)	Severe	Severe	Severe	Mild	Normal	Normal	Normal	Normal
Vestibular defect	Normal	Normal	Normal	Normal	Normal	Normal	Normal	Normal
BCVA (R/L)	HM/20 cm FC/15 cm	0.1/0.05	0.12/0.2	1.0/1.0	1.0/1.0	1.0/1.0	1.0/1.0	1.0/1.0
ERG findings	Extinguished rod and cone	NA	Extinguished rod and cone	Slightly reduced rod and cone	Normal	NA	Normal	Normal
Fundus	RP	NA	RP	Macular lesions	Normal	NA	Normal	Normal
OCT	Reduce in thickness of the photoreceptor layer	NA	Reduce in thickness of the photoreceptor layer	Discontinuity of IS/OS junction line	Normal	NA	Normal	Normal
Comments	Wearing hearing aids	No	Wearing hearing aids, IOL both eyes	No	No	No	No	No

BCVA best-corrected visual acuity; ERG electroretinograms; Y years; FC finger count; F female; HM hand movement; IOL intraocular implantation; IS/OS inner/outer segment; L left eye; M male; NA not available; OCT optical coherence tomography; R right eye.

### Clinical characteristics of the USH2 families

**Figure 3:**
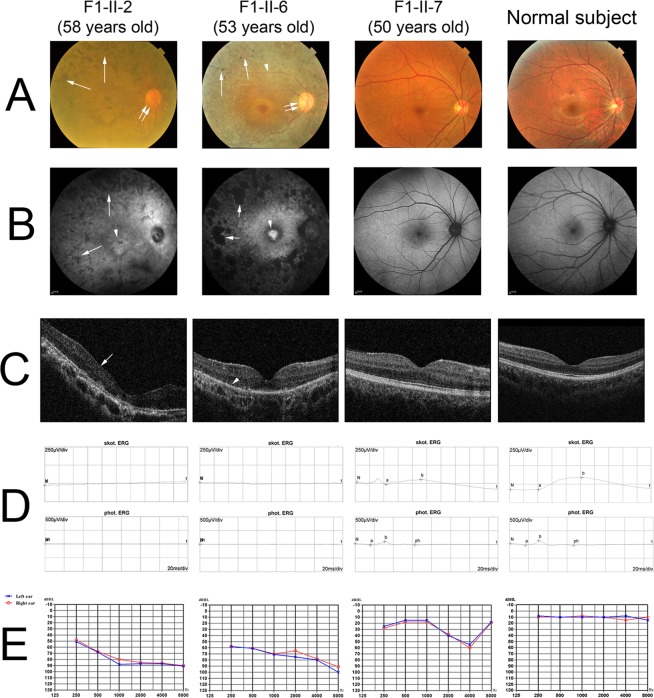
Clinical characteristics of the recruited Chinese retinitis pigmentosa family 1. (**A**) The fundus photographs of the affected and unaffected members of family F1 showed the attenuation of retinal arteries (arrow head), the bone-spicule pigmentation of the retina (arrows) and the waxy optic disc (double arrows). (**B**) Patchy hypo-autofluorescence (arrows) was observed in the mid-peripheral retina and hyper-autofluorescence in the macula (arrow head) by auto-fluorescence imaging. (**C**) OCT imaging displayed significant reduction in retinal thickness (arrow) and extensive disruption in ellipsoid zone (arrow head). (**D**) The representative flattening in the rod and cone ERG response was detected. (**E**) The PTA results showed bilateral down-sloping severe sensorineural hearing loss.

All the 4 recruited families initially diagnosed with RP followed the pattern of autosomal recessive inheritance (Fig. 1). For the four-generation family F1, the three affected patients (F1-II-2, F1-II-4 and the proband: F1-II-6) presented with the onset of night blindness at teenage and typical RP features. The fundus photographs showed the attenuation of retinal arteries, the bone-spicule pigmentation of the retina and the waxy optic disc (Fig. 3A). Patchy hypo-autofluorescence was observed in the mid-peripheral retina and hyper-autofluorescence in the macula by auto-fluorescence imaging (Fig. 3B). Moreover, optical coherence tomography (OCT) imaging displayed significant reduction in retinal thickness and extensive disruption in ellipsoid zone (Fig. 3C). The representative flattening in the rod and cone ERG response was detected (Fig. 3D). The pure-tone audiometry (PTA) results showed bilateral down-sloping severe sensorineural hearing loss (Fig. 3E), indicating the diagnosis of USH2. From the medical history, the affected members did not exhibit the delayed gait development, poor speech acquisition and balance disturbance or unstable walking. Their unaffected brother (F1-II-7) showed normal gross motor development, speech ability and eyesight; yet, he has bilateral mild sensorineural hearing loss and mild reduction in cone and rod response (Table 2). The remaining unaffected subjects (F1-III-5, F1-III-8, F1-III-9, F1-III-11) did not show any symptoms or abnormalities in the clinical examinations.

The three probands (F2-II-11, F3-II-6 and F4-II-15) from the other three recruited families showed progressive night blindness and mild to severe hearing impairment, which indicated the diagnosis of USH2. The fundus abnormalities, the flattening in the rod and cone responses ERG and the reduced photoreceptor layer thickness in OCT examination supported the typical RP features in these patients.

### *USH2A* mutation analysis in sporadic RP patients

To further extend the *USH2A* mutation analysis, the *USH2A* gene variants was searched from 118 sporadic RP patients with the WES analysis. Total of 25 candidate variants were identified in 23 sporadic RP patients. Among these 23 sporadic RP patients, 13 of them carried only 1 *USH2A* variant, and 10 patients carried two or three variants in the *USH2A* gene. For these 10 sporadic RP patients, they showed progressive night blindness and mild to severe hearing impairment, indicating the diagnosis of USH2. For the 16 *USH2A* variants identified in the 10 sporadic RP (USH2) patients, 12 of them have been reported and 4 were novel (Table 3). Combining the heterozygous carrier and the compound heterozygous patients, 8 novel variants were discovered from 23 sporadic RP patients in this study. The novel variants have been verified by Sanger sequencing and were not found in our 200 control subjects.

**Table 3:** The *USH2A* variants identified in sporadic RP patients.

Sporadic patients	Age (yrs)	Gender	BCVA (R/L)	Ophthalmic examinations	Diagnosis	*USH2A* Mutation	SIFT	Polyphen2 HDIV	Mutation Taster	CADD	MAF	Domain
RP-P1	58	F	0.06/0.06	Positive	RP ou; IOL ou	c.9570 + 1 G > A	N/A	N/A	D^3^	15.17	5.44 × 10^−4^	/
						c.8559-2 A > G	N/A	N/A	D^3^	26.2	4.35 × 10^−4^	/
RP-P2	61	M	0.25/0.15	Positive	RP ou; IOL ou	c.12569 T > C:p.V4190A	D^1^	D^2^	D^3^	19.21	**Novel**	Fibronectin type-III
						c.5953 G > A:p.E1985K	T	D^2^	D^3^	31	6.53 × 10^−4^	Fibronectin type-III
						c.2802 T > G:p.C934W	D^1^	D^2^	D^3^	16.44	2.51 × 10^−3^	Laminin EGF-like
RP-P3	10	F	0.2/0.25	Positive	RP ou	c.9570 + 1 G > A	N/A	N/A	D^3^	15.17	5.44 × 10^−4^	/
						c.4576 G > A:p.G1526R	T	D^2^	D^3^	32	2.52 × 10^−4^	Laminin G-like
RP-P4	58	M	0.2/0.5	Positive	RP ou; IOL od	c.15178 T > C:p.S5060P	T	D^2^	N	21.9	6.02 × 10^−4^	**/**
						c.14017 T > C:p.Y4673H	T	P	D^3^	16.88	1.50 × 10^−4^	Fibronectin type-III
						c.2802 T > G:p.C934W	D^1^	D^2^	D^3^	16.44	2.51 × 10^−3^	Laminin EGF-like
RP-P5	57	F	0.3/0.4	Positive	RP ou; CC ou	c.15178 T > C:p.S5060P	T	D^2^	D^3^	21.9	6.02 × 10^−4^	/
						c.4576 G > A:p.G1526R	T	D^2^	D^3^	32	2.52 × 10^−4^	Laminin G-like
RP-P6	50	M	0.5/0.6	Positive	RP ou; IOL ou	c.15178 T > C:p.S5060P	T	D^2^	N	21.9	6.02 × 10^−4^	**/**
						c.13979 C > T:p.P4660L	D^1^	D^2^	D^3^	24	0	Fibronectin type-III
						c.206 G > T:p.S69I	D^1^	D^2^	D^3^	17.07	3.01 × 10^−4^	/
RP-P7	81	M	0.1/0.1	Positive	RP ou; IOL os; CC od	c.11233 T > C:p.Y3745H	T	D^2^	D^3^	19.24	5.01 × 10^−4^	Fibronectin type-III
						c.6998 T > C:p.V2333A	T	D^2^	D^3^	22	4.76 × 10^−3^	Fibronectin type-III
RP-P8	55	F	0.25/0.4	Positive	RP ou; CC ou	c.11261 G > T:p.G3754V	T	D^2^	D^3^	7.361	**Novel**	Fibronectin type-III
						c.9570 + 1 G > A	N/A	N/A	D^3^	15.17	5.44 × 10^−4^	/
RP-P9	34	F	0.25/0.1	Positive	RP ou; IOL os; CC od	c.5398 T > A:p.W1800R	D^1^	D^2^	D^3^	20.9	**Novel**	Laminin G-like
						c.2187 C > A:p.C729*	T	N/A	D^3^	43	2.73 × 10^−4^	Laminin EGF-like
RP-P10	48	M	0.12/0.15	Positive	RP ou; IOL os; CC od	c.13330 C > A:p.P4444T	D^1^	D^2^	D^3^	15.6	**Novel**	Fibronectin type-III
						c.2802 T > G:p.C934W	D^1^	D^2^	D^3^	16.44	2.51 × 10^−3^	Laminin EGF-like
RP-P11	41	M	0.5/0.4	Positive	RP ou; IOL ou	c.4481 A > T:p.N1494I	D^1^	D^2^	D^3^	20.9	**Novel**	**/**
RP-P12	27	M	0.02/0.15	Positive	RP ou	c.9371 + 1 G > C	N/A	N/A	D^3^	13.59	0	/
RP-P13	63	F	0.02/0.06	Positive	RP ou; IOL od; CC os	c.2409delC:p.L803fs	N/A	N/A	N/A	N/A	**Novel**	Laminin EGF-like
RP-P14	27	F	0.6/0.6	Positive	RP ou	c.6998 T > C:p.V2333A	T	D^2^	D^3^	22	4.76 × 10^−3^	Fibronectin type-III
RP-P15	46	F	0.12/0.5	Positive	RP ou	c.2802 T > G:p.C934W	D^1^	D^2^	D^3^	16.44	2.51 × 10^−3^	Laminin EGF-like
RP-P16	50	M	HM/10 cm 0.02	Positive	RP ou; CC ou	c.1181 C > G:p.P394R	D^1^	D^2^	D^3^	24.7	5.50 × 10^−5^	Laminin N-terminal
RP-P17	23	F	0.3/0.25	Positive	RP ou; IOL ou	c.5608 C > T:p.R1870W	D^1^	D^2^	D^3^	26.9	3.87 × 10^−3^	Laminin G-like
RP-P18	59	M	0.4/0.3	Positive	RP ou; IOL od; CC os	c.4382delA:p.Q1461fs	N/A	N/A	N/A	N/A	**Novel**	Fibronectin type-III
RP-P19	63	M	0.4/0.25	Positive	RP ou	c.15178 T > C:p.S5060P	T	D^2^	N	21.9	6.02 × 10^−4^	**/**
RP-P20	48	M	0.8/0.8	Positive	RP ou; IOL ou	c.9570 + 1 G > A	N/A	N/A	D^3^	15.17	5.44 × 10^−4^	/
RP-P21	55	M	0.6 CF/30 cm	Positive	RP ou; CC ou	c.7100 G > A:p.G2367E	T	D^2^	D^3^	16.81	**Novel**	Fibronectin type-III
RP-P22	56	F	0.4/0.4	Positive	RP ou; CC ou	c.8254 G > A:p.G2752R	D^1^	D^2^	D^3^	29.1	2.72 × 10^−4^	Fibronectin type-III
RP-P23	26	M	0.25/0.3	Positive	RP ou	c.9041 C > A:p.T3014N	T	D^2^	D^3^	12.81	2.46 × 10^−3^	Fibronectin type-III

AD: autosomal dominant; AR: autosomal recessive; B: benign; CC: complicated cataract; D^1^: deleterious; D^2^: damaging; D^3^: disease-causing; F: female; Hetero: heterozygous; IOL: intraocular lens; M: male; MAF: minor allele frequency in East Asian population; N: polymorphism; N/A: not available; ou: binoculus; P: possibly damaging; RP: retinitis pigmentosa; T: tolerated; yrs, years.

### Bioinformatics and clinical correlation of *USH2A* mutations

**Figure 4:**
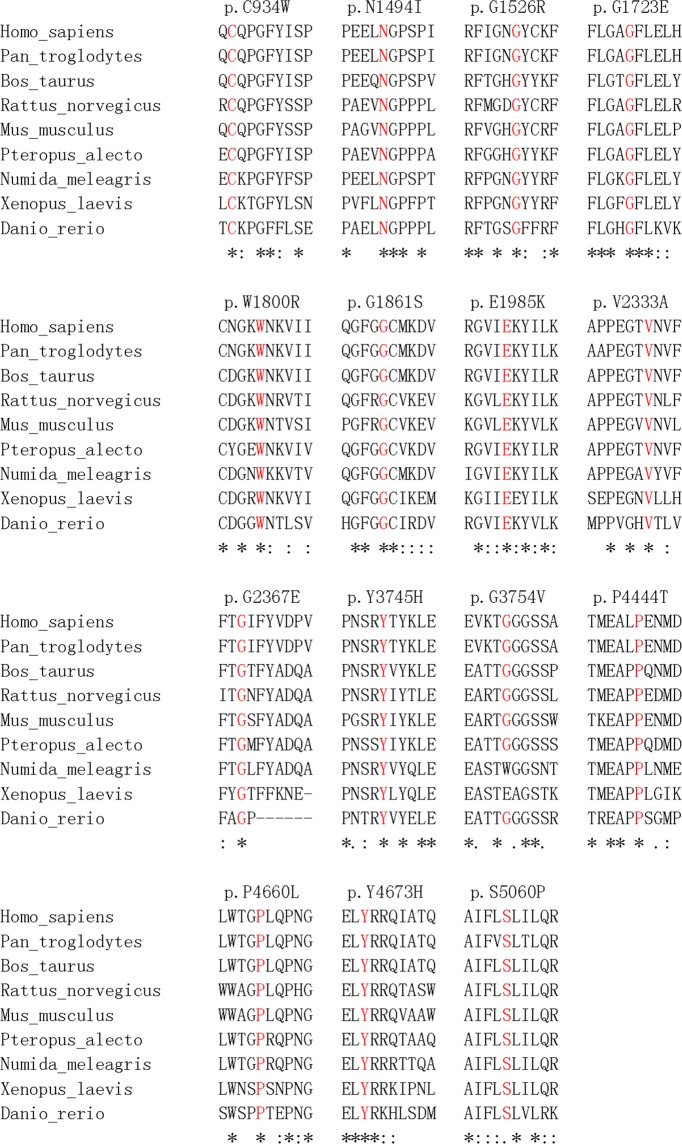
Multiple sequence alignment of *USH2A* protein across different species. *USH2A* protein sequences of *Homo sapiens*, *Pan troglodytes*, *Bos taurus*, *Rattus norvegicus*, *Mus musculus*, *Pteropus alecto*, *Numida meleagris*, *Xenopus laevis* and *Danio rerio* were aligned by Clustal Omega. The conservative of the 15 missense mutations identified in USH and sporadic RP patients were displayed.

In this study, we identified 15 missense mutations from 14 USH2 and sporadic RP patients: c.2802 T > G (p.C934W), c.A4481A > T (p.N1494I), c.4576 G > A (p.G1526R), c.5168 G > A (p.G1723E), c.5398 T > A (p.W1800R), c.5581 G > A (p.G1861S), c.5953 G > A (p.E1985K), c.6998 T > C (p.V2333A), c.7100 G > A (p.G2367E), c.11233 T > C (p.Y3745H), c.11261 G > T (p.G3754V), c.13330 C > A (p.P4444T), c.13979 C > T (p.P4660L), c.14017 T > C (p.Y4673H) and c.15178 T > C (p.S5060P). All of the amino acids are conserved in *USH2A* protein across 8 different species (Fig. 4), except G3754 (partially conserved), indicating that the missense mutations could affect the *USH2A* protein function or structure.

Compare to the missense mutations, the nonsense (c.187 C > T, p.R63*; c.2187 C > A, p.C729* and c.5079 G > A, p.W1693*), indel (c.12086dupA, p.H4029Qfs*68) and the splice site mutations (c.5858-1 G > A, c.8559-2 A > G and c.9570 + 1 G > A) would lead to drastic changes to the *USH2A* protein structure by creating premature stop codon (p.R63*: *USH2A* protein with 62 amino acids only, p.C729*: *USH2A* protein with 728 amino acids only, and p.W1693*: *USH2A* protein with 1692 amino acids only) or translational frameshifting (c.12086dupA: *USH2A* protein prematurely with 4097 amino acids, c.5858-1 G > A: affected mRNA processing for exon 30, c.8559-2 A > G: affected mRNA processing for exon 43 and c.9570 + 1 G > A: affected mRNA processing for exon 48). With references to the bioinformatics analyses, *USH2A* dysfunction is expected from a truncated protein or nonsense-mediated decay.

**Figure 5:**
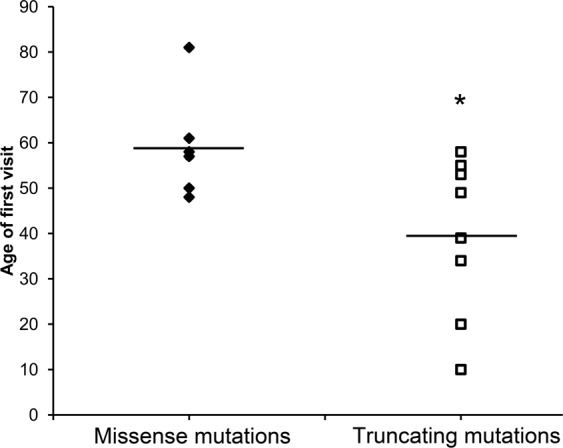
Age of first visit of USH2/RP patients with different properties of *USH2A* mutations. The 15 USH2/RP patients were divided into the missense mutation only group and the truncating mutation group (nonsense, indel or splice site mutations) based on the properties of *USH2A* mutations. The patients carried 1 or 2 nonsense, indel or splice site mutations in the protein truncational group have a younger age of first visit (39.75 ± 16.34 years) than the patients carried only the missense mutations (59.17 ± 10.76 years. *p < 0.05.

Based on the changes in protein properties by the mutations, we divided the 14 USH2 and sporadic RP patients into the missense mutation only group and the truncating mutation group. The patients carrying 1 or 2 nonsense, indel or splice site mutations in the truncating mutation group have a younger age of first visit (39.75 ± 16.34 years) than the patients only carrying the missense mutations (59.17 ± 10.76 years, *p* = 0.017; Fig. 5), implying that the nonsense, indel and splice site mutations could lead to earlier onset of disease or more severe disease progression compared to the missense mutations.

## Discussion

USH is usually discovered first by RP in the initial screening, and ophthalmic examination alone might not easily discover other invisible dysfunctions. Genetic diagnosis, in this scenario, can facilitate the precise clinical diagnosis of complicated diseases for better management and counseling. In this study, we identified 10 sporadic RP patients, out of 118 recruited subjects, carrying at least 2 compound heterozygous *USH2A* mutations (Table 3), confirming the diagnosis of USH2. This indicates that 8.47% of sporadic RP patients could belong to USH in Southern Chinese population.

Previous studies in Chinese populations have reported the identification of *USH2A* and *ADGRV1* mutations for USH2 in Chinese populations^20,22–24^. The *USH2A* gene, located on chromosome 1q41, is consisted of 72 exons, whereas the *ADGRV1* gene, located on chromosome 5q14.3, is consisted of 90 exons. It is not efficient and time-consuming to screen the disease-causing mutations in these genes by traditional Sanger sequencing. Target exome sequencing has been applied to map the disease-causing gene for USH^20,22–24^. Yet, the WES analysis, based on the general enrichment and capturing platform, can efficiently delineate the variants in all genes with a reasonable price and processing time. It is a favorable sequencing analysis for the disease-causing mutation screening in the inherited retinal dystrophies^20,25,26^.

USH is an autosomal recessive disorder characterized by retinitis pigmentosa (RP) and bilateral sensorineural hearing loss. In family F1, the *USH2A* mutation carrier (F1-II-7; c.187 C > T p.R63*) had best corrected visual acuity of 1.0, showed normal gross motor development as well as speech ability and did not complained of nyctalopia; yet, he has bilateral mild sensorineural hearing loss and slightly reduction in rod and cone ERG responses (Fig. 3 and Table 2). The bilateral mild sensorineural hearing loss on PTA could probably be due to aging. However, whether the single nonsense mutation would lead to ERG response reduction or a milder symptoms of USH requires further investigations. Yet, we identified 21 sporadic RP patients carrying single *USH2A* variant, and whether carrying single *USH2A* variant contributes to the RP development is also unknown.

Usherin, coded by the *USH2A* gene, is a basement membrane protein with 5202 amino acids. The first 31 amino acids are responsible for the signal peptide, and the rest of the protein is composed of the N-terminal laminin domain, the laminin EGF-like domains, the laminin G-like domains, the fibronectin type-III domains, a transmembrane domain and a cytoplasmic tail. In this study, we identified 30 mutations in the *USH2A* gene, of which p.C934W and c.9570 + 1 G > A in 5 patients (18.52%) and p.S5060P in 4 patients (14.81%), indicating that these mutations have higher frequencies in Chinese populations and a potential of founder effect. In the 14 USH2 and sporadic RP patients, we identified 15 missense mutations from by the WES analysis (Tables 1 and 3). One mutation (p.C934W) is located in the laminin EGF-like domain, 3 mutations (p.G1526R, p.G1723E and p.W1800R) in the laminin G-like domains and 7 mutations (p.E1985K, p.V2333A, p.Y3745H, p.G3754V, p.P4444T, p.P4660L and p.Y4673H) in the fibronectin type-III domains and 3 mutations (p.N1494I, p.G1861S and p.S5060P) in the linker regions, indicating that they could induce structural or functional changes to the usherin protein. Moreover, the p.C934W mutation also belong to the site for disulphide bond formation. The amino acids of these 14 missense mutations, except p.G3754V (partially conserved), are highly conserved across 8 different species (Fig. 4), reassuring the importance of these amino acid residues for usherin protein function. Compared to the missense mutations, the nonsense (p.R63*, p.C729* and p.W1693*), indel (c.12086dupA) and the splice site mutations (c.5858-1 G > A, c.8559-2 A > G and c.9570 + 1 G > A) would lead to a truncated *USH2A* protein by the premature stop codon (p.R63*: *USH2A* protein with 62 amino acids only, p.C729*: *USH2A* protein with 728 amino acids only, and p.W1693*: *USH2A* protein with 1692 amino acids only), translational frameshifting (c.12086dupA: *USH2A* protein with 4097 amino acids, c.5858-1 G > A: affected mRNA processing for exon 30, c.8559-2 A > G: affected mRNA processing for exon 43 and c.9570 + 1 G > A: affected mRNA processing for exon 48) or reduced *USH2A* expression by nonsense-mediated decay. Accordingly, the patients carried the nonsense, indel or splice site mutations have a significant younger age of first visit (39.75 ± 16.34 years) than the patients carried only the missense mutations (59.17 ± 10.76 years; Fig. 5), implying that the nonsense, indel and splice site mutations could lead to earlier onset of disease or more severe disease progression compared to the missense mutations. Similarly, it was reported that USH2 patients carrying the p.Glu767Serfs*21 *USH2A* mutation can have earlier diagnosis of disease, onset of night blindness, onset of visual field loss, diagnosis of cataract and onset of hearing loss than those carrying the p.Cys759Phe mutation^27^. Moreover, patients with two truncating *USH2A* mutations develop significantly more severe hearing impairment throughout life than patients with two non-truncating mutations^28^. Yet, how these mutations cause visual defects and hearing impairment requires further functional and mechanism investigations.

In summary, this study revealed 11 novel variants in *USH2A* gene from four unrelated Chinese USH2 families and 23 sporadic RP patients, expanding the mutation spectrums of *USH2A* gene. Ten sporadic RP patients were diagnosed as USH2 after WES. Genetic diagnosis is an adequate strategy to aid the clinical diagnosis for better clinical management and counseling. It should be recommended as a routine examination for inherited retinal dystrophies.

## Materials and Methods

### Study subjects and clinical examinations

Four unrelated Chinese families initially diagnosed with RP were recruited from the Joint Shantou International Eye Center of Shantou University and the Chinese University of Hong Kong, including seven patients and nine unaffected family members (Fig. 1). The audiological criteria to consider a USH2 is mild to moderate sensorineural hearing loss^29^. Comprehensive ophthalmic examinations, including best-corrected visual acuity (BCVA), fundus photography, visual field tests, OCT, electroretinogram (ERG) and auto-fluorescence, were performed for all patients and some of the unaffected family members. The severity of hearing loss was evaluated by PTA and classified as mild (25–40 dB), moderate (41–70 dB), severe (71–90 dB), profound (>90 dB). Vestibular function was recorded from the detailed medical history, including motor development, speech acquisition, stability and balance.

For the validation analysis, 118 sporadic RP patients and 200 unrelated control subjects were also enrolled. The sporadic RP patients generally showed pubertal night blindness, restricted peripheral vision, progressive vision loss, overall bone-spicule pigmentation of the retina, attenuation of retinal vessels, and the flattening of the rod and cone ERG responses. The control subjects, aged 60 years or above, did not have any family history or symptom of inherited retinal diseases, hearing loss, or any other major eye diseases except mild senile cataracts and low myopia.

Peripheral blood was collected from the study subjects, and the genomic DNA was extracted by TIANGEN DNA blood Kit DP318 (TIANGEN, Beijing, China), and stored at −80 °C freezer before sequencing analysis.

This study protocol was approved by the Ethics Committee on Human Research at the Joint Shantou International Eye Center of Shantou University and the Chinese University of Hong Kong, which is in accordance with the tenets of the Declaration of Helsinki. Written informed consents were obtained from all study subjects or their guardians after explanation of the nature and possible consequences of the study.

### Whole exome sequencing analysis

WES analysis was conducted for each proband from the recruited families and 118 sporadic RP patients. Briefly, genomic DNA was fragmented into 150–200 bp DNA libraries by S2/E210 Focused-ultrasonicator (Covaris, Woburn, MA). All coding exons were captured by and hybridized to a customized array (Agilent Technologies, Santa Clara, CA). The purified and enriched DNA libraries were sequenced using the HiSeq2500 platform (Illumina, San Diego, CA) to generate paired-end reads for 90 cycles per reads. The results were aligned to human genome reference (UCSC hg19) in the National Center for Biotechnology Information (NCBI) database using Burrows Wheeler Aligner Multi-Vision software package (BWA) for unique mapped reads. The single nucleotide polymorphisms (SNPs) and insertion/deletion variants (indels) were identified using the SAMtools and BCFtools. The detected variants were annotated with ANNOVAR with reference to the following databases for further variant filtering, including 1000 Genome Project (http://www.internationalgenome.org/), gnomAD (http://gnomad-old.broadinstitute.org/), EXAC (http://exac.broadinstitute.org/), and dbSNP (http://www3.ncbi.nlm.nih.gov/SNP/).

To ensure the accuracy and efficacy of the candidate mutations, the synonymous, intergenic and intronic variants were first filtered out. Those variants with minor allele frequency (MAF) lower than 1% or absent in East Asian population from gnomAD and EXAC were reserved. The candidate variants were compared to the reported RP causative genes in RentNet (http://sph.uth.edu/retnet/). The retained variants were predicted by the online bioinformatics software, including SIFT (http://sift.jcvi.org/), PolyPhen-2 (http://genetics.bwh.harvard.edu/pph2/), mutationtaster (http://www.mutationtaster.org/), CADD (http://cadd.gs.washington.edu/), ExPASy (https://web.expasy.org/translate/) and Clustal Omega (https://www.ebi.ac.uk/Tools/msa/clustalo/).

### Sanger sequencing validation

The potential causative gene variants identified by WES in the Chinese RP families were validated by polymerase chain reaction (PCR) and Sanger sequencing with specific primers (AIJI Biotechnology Company, Guangdong, China; Supplementary Table 1) in the ABI 3730XL Genetic Analyzer (Applied Biosystems, USA) to confirm the co-segregation pattern. The sequencing results were analyzed by NOVOSNP (http://www.molgen.vib-ua.be/bioinfo/novosnp/) with the alignment to the reference DNA sequence. In addition, the identified variants were also screened in 200 control subjects.

### Statistical analysis

Independent T-test was used to compare the means between the study groups with different properties of *USH2A* mutations. All statistical analyses were performed by the commercially available software (IBM SPSS Statistics 22; SPSS Inc., Chicago, IL). Significance was defined as *p* < 0.05.

## Supplementary information

Supplementary Table and Figure
