# Supplementary material for: Whole exome sequencing identifies novel USH2A mutations and confirms Usher syndrome 2 diagnosis in Chinese retinitis pigmentosa patients
Source: Sci Rep. 2019 Apr 4;9:5628. doi: 10.1038/s41598-019-42105-0 (PMC6449333; doi:10.1038/s41598-019-42105-0)
Supplement: Supplementary file 1 — Supplementary Table and Figure [file 41598_2019_42105_MOESM1_ESM.docx]

**Whole exome sequencing identifies novel *USH2A* mutations and confirms Usher syndrome 2 diagnosis in Chinese retinitis pigmentosa patients**

Tsz Kin Ng,^1,2,3,*^ Wenyu Tang,^1,*^ Yingjie Cao,^1^ Shaowan Chen,^1^ Yuqian Zheng,^1^ Xiaoqiang Xiao,^1^ Haoyu Chen.^1^

^1^ Joint Shantou International Eye Center of Shantou University and The Chinese University of Hong Kong, Shantou, Guangdong, China

^2^ Shantou University Medical College, Shantou, Guangdong, China

^3^ Department of Ophthalmology and Visual Sciences, The Chinese University of Hong Kong, Hong Kong

^*^ These authors contribute equally to this work.

Correspondence:

Prof. Haoyu Chen, MD

Joint Shantou International Eye Center of Shantou University and The Chinese University of Hong Kong, North Dongxia Road, Shantou, Guangdong, China 515041

Email: drchenhaoyu@gmail.com; Phone: +86-754 88393560; Fax: +86-754 88393560

**Supplementary Table 1:** Primers for the novel *USH2A* variant verification by Sanger sequencing

|  | **Gene** | | **Mutations** | **Forward primer sequence (5'>3')** | **Reverse primer sequence (5'>3')** | **Product size (bp)** | **Tm (^o^C)** |
| --- | --- | --- | --- | --- | --- | --- | --- |
| **F1** | *USH2A* | NM_206933:c.15427C>T:p.R5143C | | CCGATACCAAAATTCCCCGG | GCATTCGGTGAAGTACAGGC | 299 | 62.0 |
|  | *USH2A* | NM_206933:c.12086dupA:p.H4029fs | | CTCTGCTGTAGTGTTTGCGC | CACTGTAGTAGCAATGCCCG | 322 | 61.6 |
|  | *USH2A* | NM_206933:c.187C>T:p.R63* | | TGTTGATCTTTGCCTATTTTGCT | TCTTGTCTGGTGTGATGCAG | 285 | 61.6 |
| **F2** | *PDE6B* | NM_000283:c.145G>T:p.D49Y | | CCGGGGTTCCTAATCTCACT | TCCTCGACGTTCACCATCTT | 561 | 56.4 |
|  | *USH2A* | NM_206933:c.5581G>A:p.G1861S | | GCGGAATTTAGAAAATGTGGGC | ACCCTCGTAAACACTCTGCT | 481 | 62.0 |
|  | *USH2A* | NM_206933:c.9570+1G>A | | TGGTATCCATGCGCTAAAACTC | GCATCCTTTCTTTTCCGTGGA | 266 | 62.0 |
|  | *USH2A* | NM_206933:c.15427C>T:p.R5143C | | CCGATACCAAAATTCCCCGG | GCATTCGGTGAAGTACAGGC | 299 | 62.0 |
| **F3** | *EYS* | NM_001292009:c.2980C>G:p.P994A | | AGGTTTCTATTTGCTTGCAGACA | ATGAGTGAGAACATGCGGTG | 577 | 62.0 |
|  | *KIAA1549* | NM_020910:c.5653C>T:p.R1885W | | ACAGTGACGTTTGCCATGTT | GGTGCTCTGTTTCTGGGAGA | 260 | 55.5 |
|  | *PRPH2* | NM_000322:c.367C>T:p.R123W | | TGGGAAGATCTGCTACGACG | TGAATCTCAAACCAGTCCCGA | 300 | 55.5 |
|  | *USH2A* | NM_206933:c.5079G>A:p.W1693* | | ACTATAGCTGATGGTGTGGTG | TGTGCACCATTGGAATAACTAAG | 484 | 62.0 |
|  | *USH2A* | NM_206933:c.5168G>A:p.G1723E | | GCCCATCACTGCCTCCTATA | CAGCAAGAAAATCAGGTCCATCT | 593 | 62.0 |
| **F4** | *CNGB1* | NM_001297:c.2681G>A:p.R894H | | CCCGTGGTCTGTGTGAAGT | CGTGACCATCTTACCCAGCA | 273 | 62.0 |
|  | *USH2A* | NM_206933:c.2802T>G:p.C934W | | GCAACTGTGATAAGACTGGGAC | CCACAAACCAGAAACAGGGAG | 356 | 62.0 |
|  | *USH2A* | NM_206933:c.5858-1G>A | | TCGCTTAAACTGAATGGCTCA | CCTGTGAGGTTGCTTGTATTGA | 380 | 61.6 |
| **RP-P8** | *USH2A* | NM_206933:c.11261G>T:p.G3754V | | AGTCAAGTTTCTGGAAAGGGG | TGTTTACACACACACACACATAC | 339 | 60.9 |
| **RP-P11** | *USH2A* | NM_206933:c.4481A>T:p.N1494I | | GTGTTTTCAGTTCCTAGAGCCA | CTGGGTGAGTGGAGCTGG | 370 | 59.5 |
| **RP-P15** | *USH2A* | NM_206933:c.2409delC:p.L803fs | | GGCATTGCTTGTGAGAAAACA | AAGGCAGACAGAGGAAAGAATT | 500 | 60.9 |
| **RP-P25** | *USH2A* | NM_206933:c.4382delA:p.Q1461fs | | CACACTGCTAAATCCCAAGAACT | AGCAATCAGAGTTAGTGAGGGA | 264 | 61.6 |
| **RP-P29** | *USH2A* | NM_206933:c.7100G>A:p.G2367E | | GTGCTTTGATCCTGCTGACA | AACCACATTCTGAGAACCGT | 495 | 59.5 |

**
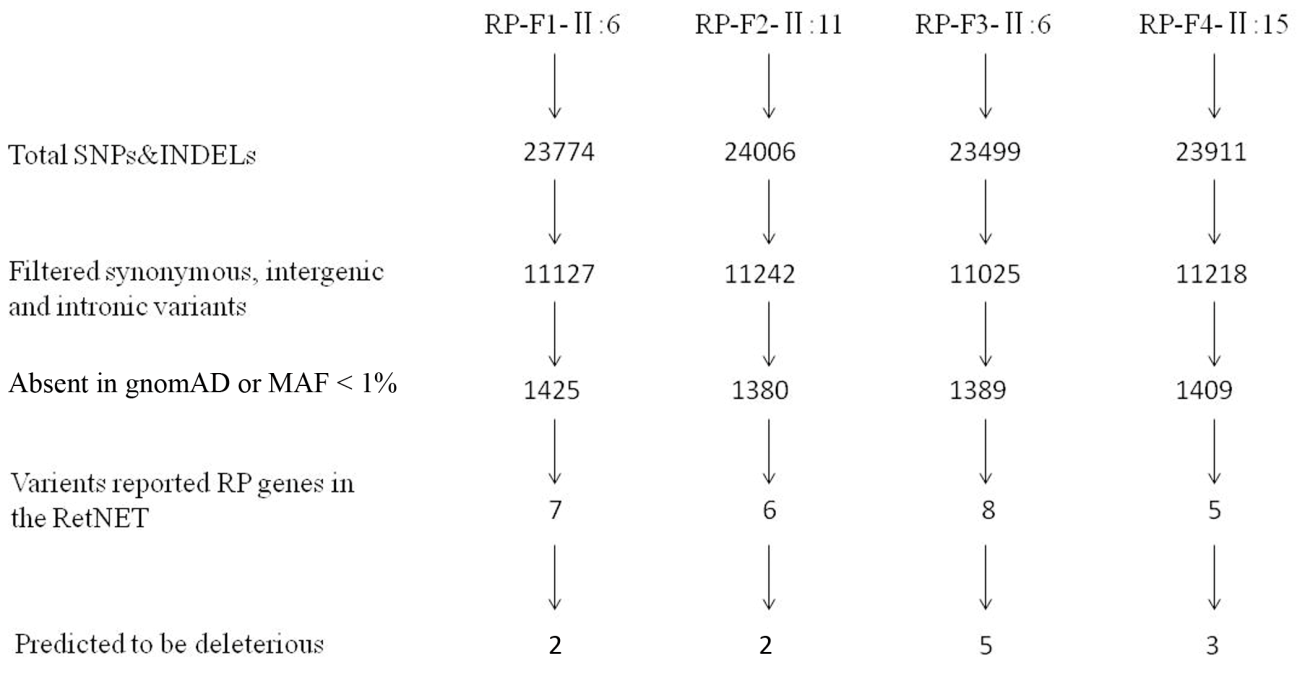
**

**Supplementary Figure 1: Whole exome sequencing analysis of the recruited Chinese retinitis pigmentosa families.**

Filtering procedures of the whole exome sequencing analysis for the 4 recruited USH families. The total numbers of variants of exons and splice sites identified were 23,774 for F1-II-6, 24,006 for F2-II-11, 23,499 for F3-II-6 and 23,911 for F4-II-15. After filtering the synonymous, intergenic, intronic and common variants, the candidate variants of known RP genes were reduced to 2 for F1-II-6, 2 for F2-II-11, 5 for F3-II-6 and 3 for F4-II-15.
